# Self-care related knowledge, attitude, practice and associated factors among patients with diabetes in Ayder Comprehensive Specialized Hospital, North Ethiopia

**DOI:** 10.1186/s13104-019-4072-z

**Published:** 2019-01-18

**Authors:** Haftom Niguse, Goitom Belay, Girmatsion Fisseha, Tesfaye Desale, Goitom Gebremedhn

**Affiliations:** 1 School of Pharmacy, College of Health Sciences, Mekelle University, Mekelle, Ethiopia; 2 School of Public Health, College of Health Sciences, Mekelle University, Mekelle, Ethiopia; 3 Tigray Health Research Institute, Tigray Regional Health Bureau, Mekelle, Ethiopia

**Keywords:** Self-care, Diabetes, Knowledge, Attitude, Practice, Ayder Comprehensive Specialized Hospital, Ethiopia

## Abstract

**Objective:**

A good self-care practice is important for patients with diabetes to achieve the desired treatment targets and to contribute meaningfully in the management of their disease. The study aimed to assess the level of knowledge, attitude and practice of diabetes self-care and to identify the factors associated with diabetes self-care.

**Results:**

A total of 338 patients with diabetes having mean age of 45.8 years were included in the study. Among those 70.4%, 70.4% and 25.5% of the patients had a good knowledge, attitude and self-care practices, respectively. Being male (AOR = 2.7, 95% CI 1.30–5.65), living in urban (AOR = 3.37, 95% CI 1.39–8.15) and earning medium income (AOR = 2.55, 95% CI 1.15–5.65) were significantly associated with having good knowledge of self-care while being widowed (AOR = 0.15, 95% CI 0.03–0.70) was associated with having poor knowledge. Having a higher income (AOR = 7.95, 95% CI 1.54–41.12) was significantly associated with a good attitude towards diabetic self-care. However, taking both insulin and oral hypoglycemics (AOR = 0.06, 95% CI 0.01–0.67) was associated with a poor attitude. Being Muslim (AOR = 3.14, 95% CI 1.28–7.91), living in urban areas (6.47, 95% CI 1.38–30.43) and earning high income (AOR = 3.03, 95% CI 1.10–8.35) were determinant of good self-care practice. Efforts should be made to improve self-care practices of patients in closing the gap between knowledge and practice.

**Electronic supplementary material:**

The online version of this article (10.1186/s13104-019-4072-z) contains supplementary material, which is available to authorized users.

## Introduction

Diabetes has a tremendous impact on the patients’ quality of life and productivity. It is a leading cause of acquired blindness, kidney failure and lower leg amputations. Worldwide, there are more than 450 million patients with diabetes with a majority (75%) of adults live in low and middle income countries [1–3]. According to the International Diabetes Federation (IDF) report, there were 2,567,900 cases of diabetes in Ethiopia in 2015 making the adult prevalence to be 5.2% [4]. The overall prevalence of diabetes in adults in some cities of the country reaches to 6.5% [5] with 5.1% in urban and 2.1% in rural dwellers [6].

Patient knowledge regarding disease and self-care practices are found to be important for patients to achieve the desired treatment targets and contribute meaningfully in the management of their disease [7]. The chronic nature of diabetes and handling of the majority of the day to day care of the patient in ambulatory care necessities to promote and strengthen self-care practices among all patients with diabetes [7, 8].

Though, there was significant variation across countries, self-care behaviour on diabetes is less than optimal in all countries especially in the developing world [7]. Only 46% of patients with Type 1 and 39% of patients with type 2 diabetes practiced in at least two-thirds of their self-care domains in Ethiopia [9]. Most studies are done on patients with type 2 diabetes and little is known about the factors associated with self-care practice of patients with diabetes in Ethiopia. Therefore, this study aimed to assess the knowledge, attitude and practice and the associated factors of diabetes self-care among diabetes patients attending Ayder Comprehensive Specialized Hospital.

## Main text

### Study area and period

The study was conducted at the diabetic clinic of Ayder Comprehensive Specialized Hospital, which is located in the northern Ethiopia, Tigray region, Mekelle city. The study was conducted from December 10, 2016 to January 10, 2017.

### Study design

A hospital based cross-sectional study design was utilized.

### Source population

The source population was adult patients with diabetes who visited Ayder Comprehensive Specialized Hospital.

### Study population

The study population was all adult patients with diabetes who had follow-up on a diabetic clinic of Ayder Comprehensive Specialized Hospital during the data collection period.

### Eligibility criteria

Patients with diabetes aged 18 years and above, who had a regular follow-up for at least 6 months were included. Patients with talking and hearing impairment were excluded.

### Sample size determination

The sample size was calculated by considering single population proportion formula by assuming 95% confidence interval, 1.96 standard normal variable (z score) with a 5% margin of error. By adjusting the total number of patient 2000 patients and 5% contingency it become 338 patients.

### Sampling technique

Every sixth patient was selected using systematic random sampling technique.

### Data collection instrument and techniques

Data were collected using face to face interview by clinical nurses after receiving training on how to collect the data. To maintain the validity of the data collection tool, the questionnaire was developed from the standard and translated into Tigrigna and translated back to English. The tools contained information on socio-demographics, clinical characteristics, knowledge questions developed from existing validate questionnaires in the ‘spoken knowledge in low literacy in diabetes knowledge assessment scale’ (SKILLDs) [10], the attitude questions developed from the Diabetes Attitude Survey (DAS3) [11] University of Michigan Diabetes Research and Training Center and self-care practice questions adapted from the summary of diabetic self-care activities (SDSCA) (Additional file 1) [12].

### Data processing and Analysis procedures

Data was entered, coded and analysed using SPSS version 21. Bivariate logistic regression was conducted to identify determinants of attitude, knowledge and practice of self-care. To avoid missing important factors purposively selected variables with P-value < 0.25 were entered in the multivariable logistic analysis. In multivariate logistic regression variables with P-value < 0.05 were considered as statistically significant (Additional file 2).

### Study variables

#### Dependent variables

Level of self-care knowledge, attitude and practice.

#### Independent variables

*Socio-demographic factors* Age, sex, religion, educational status, marital status, monthly income.

*Clinical characteristic* Duration of DM, DM type, comorbidity and treatment modalities.

#### Operational definition

*Knowledge* Respondents who score 50% or less were considered as having placed in the ‘poor knowledge’, while those who scored greater than 50% were considered as having ‘good knowledge’ [10].

*Attitude* Respondents who score less than 50% considered as having ‘poor attitude’ for self-care and respondents who scored 50% and above as having a ‘good attitude’ [11].

*Practice* Participants were asked about how many of the last 7 days they participated in each of the activities, scores ranged from 0 to 7. For each question a score of 5–7 was considered as good self-care and 0–4 as poor self-care practice [12].

## Results

A total of 338 adult patients with diabetes were interviewed with a response rate of 100%. The (mean ± SD) age of the participants was (45.8 ± 14.72) years and 54.4% of them were male. Most of them (89.3%) were Orthodox Christian follower. Three quarters of the respondents attended formal education. The majority (70%) of the participants were patients with type 2 diabetes. The median duration of illness among them was 6 years with IQR of 7. About half of the patients (50.6%) have been on anti-diabetic treatment for less than 5 years while 53.6% and 43.5% were on insulin injection and on oral anti-diabetics, respectively (Additional file 3).

### Factors affecting the knowledge of self-care among patients with diabetes

Among all respondents, 238 (70.4%) had good knowledge by answering > 5 correct questions out of the total 10 knowledge questions (Additional file 4).

Male patients had higher odds (AOR = 2.7, 95% CI 1.30–5.65) of having a good knowledge of self-care practice compared to female patients. Similarly, patients who live in urban areas had more than three times the odds of having a good knowledge of self-care (AOR = 3.37, 95% CI 1.39–8.15) as compared to patients who live in rural areas. Medium income patients have more than twice the odds of having good knowledge status when compared to low income patients (AOR = 2.55, 95% CI 1.15–5.65). On the other hand, widowed patients were 85% less likely to have a good knowledge status when compared to single patients (AOR = 0.15 95% CI 0.03–0.70) (Table 1).

**Table 1:** Factors affecting knowledge of self-care among patients with diabetes at Ayder Comprehensive Specialized Hospital, Mekelle, Tigray, Ethiopia, 2017

Variable	Knowledge status		COR, P-value	AOR, P-value
	Good N (%)	Poor N (%)		
Age in years				
18–35	72 (79.1)	19 (20.9)	1	1
36–50	95 (76)	30 (24)	0.84 (0.44–1.60), P < 0.59	1.57 (0.46–5.37), P < 0.47
51–65	55 (62.5)	33 (37.5)	0.44 (0.23–0.86), P < 0.02	1.16 (0.26–5.15), P < 0.85
66 and above	16 (47)	18 (531)	0.24 (0.10–0.54), P < 0.001	0.39 (0.07–2.10), P < 0.27
Sex				
Female	86 (55.8)	68 (44.2)	1	1
Male	152 (82.6)	32 (17.4)	3.76 (2.29–6.17), P < 0.00	2.71 (1.30–5.65), P < 0.01**
Level of education				
No formal education	40 (43.9)	51 (46.1)	1	1
Primary school	65 (67.7)	31 (32.3)	2.67 (1.47–4.85), P < 0.001	1.06 (0.47–2.39), P < 0.88
Secondary school	48 (85.7)	8 (14.3)	7.65 (3.25–17.99), P < 0.00	2.21 (0.74–6.57), P < 0.15
Higher school	85 (89.5)	10 (10.5)	10.84 (4.99–23.52), P < 0.00	2.68 (0.95–7.57), P < 0.06
Residence				
Urban	204 (76.7)	62 (23.3)	3.68 (2.13–6.33), P < 0.00	3.37 (1.39–8.15), P < 0.01**
Rural	34 (47.2)	38 (52.8)	1	1
Type of DM				
Type 1	78 (76.5)	24 (23.5)	1	1
Type 2	160 (67.8)	76 (32.2)	0.65 (0.38–1.10), P < 0.11	1.20 (0.38–3.79), P < 0.75
Monthly income				
Low	94 (58.4)	67 (41.6)	1	1
Medium	90 (87.4)	13 (12.6)	4.94 (2.55–9.55), P < 0.00	2.55 (1.15–5.65), P < 0.02**
High	33 (89.2)	4 (10.8)	5.88 (1.99–17.38), P < 0.001	1.75 (0.47–6.51), P < 0.40
Marital status				
Single	47 (87)	7 (13)	1	1
Married	166 (73.8)	59 (26.2)	0.42 (0.18–0.98), P < 0.44	0.45 (0.14–1.47), P < 0.17
Divorced	11 (57.9)	8 (42.1)	0.21 (0.06–0.69), P < 0.01	0.40 (0.07–2.18), P < 0.29
Widowed	14 (35)	26 (65)	0.08 (0.03–0.22), P < 0.00	0.15 (0.03–0.70), P < 0.02**
Co-morbidities				
Absent	164 (77)	49 (23)	1	1
Present	74 (59.2)	51 (40.8)	0.43 (0.27–0.70), P < 0.001	0.64 (0.29–0.39), P < 0.26
Duration of diabetes				
1–5 years	127 (76.5)	39 (23.5)	1	1
6–10 years	58 (63.7)	33 (36.3)	0.54 (0.31–0.94), P < 0.03	0.74 (0.34–1.58), P < 0.43
11–15 years	36 (66.7)	18 (33.3)	0.61 (0.31–1.20), P < 0.15	0.87 (0.34–2.21), P < 0.78
16 and above	17 (62.9)	10 (37.1)	0.52 (0.22–1.23), P < 0.14	0.85 (0.23–3.18), P < 0.81

** Shows statistically significant association

### Factors affecting the attitude of patients with diabetes on self-care practice

About 70.4% of the total patients had a good attitude towards self-care practices (Additional file 5). Patients with high monthly income were significantly associated with good attitude. On the contrary, patients who were taking both insulin and oral anti-diabetics were 94% (AOR = 0.06, 95% CI 0.01–0.67) less likely to have a good attitude when compared to patients taking insulin injection (Table 2).

**Table 2:** Factors affecting the attitude of self-care among patients with diabetes at Ayder Comprehensive Specialized Hospital, Mekelle, Tigray, Ethiopia, 2017

Variable	Attitude level		COR, P-value	AOR, P-value
	Poor	Good		
Level of education				
No formal education	42 (46.1)	49 (53.9)	1	1
Primary school	29 (30.2)	67 (69.8)	1.98 (1.09–3.61), P < 0.03	1.13 (0.53–2.40), P < 0.75
Secondary school	16 (28.6)	40 (71.4)	2.14 (1.05–4.36), P < 0.04	1.03 (0.42–2.54), P < 0.95
Higher school	13 (13.7)	82 (86.3)	5.41 (2.64–11.06), P < 0.00	1.77 (0.70–4.48), P < 0.23
Residence				
Urban	64 (24)	202 (76)	3.16 (1.84–5.42), P < 0.00	1.76 (0.84–3.69), P < 0.13
Rural	36 (50)	36 (50)	1	1
Type of DM				
Type 1	36 (35.3)	66 (64.7)	1	1
Type 2	64 (27.1)	172 (72.9)	1.47 (0.89–2.41), P < 0.13	1.15 (0.54–2.47), P < 0.71
Current medication				
Insulin injection	61 (33.7)	120 (66.3)	1	1
Oral anti-diabetic drugs	33 (22.3)	115 (87.7)	1.77 (1.08–2.91), P < 0.02	1.45 (0.70–3.04), P < 0.32
Both	6 (66.7)	3 (32.3)	0.25 (0.06–1.05), P < 0.06	0.06 (0.01–0.67), P < 0.02**
Monthly income				
Low	58 (36)	103 (64)	1	1
Medium	19 (18.4)	84 (81.6)	2.49 (1.38–4.50), P < 0.003	1.84 (0.94–3.59), P < 0.075
High	2 (5.4)	35 (94.6)	9.85 (2.29–42.47), P < 0.002	7.95 (1.54–41.12), P < 0.01**

** Shows statistically significant association

### Factors affecting the self-care practice of patients with diabetes

Among the study participants 81 (25.5%) of them had a good diabetes self-care practice (Additional file 6). Muslim patients were more likely to adhere the self-care practice (AOR = 3.14, 95% CI 1.28–7.91). Similarity patients with high monthly income were more likely to have a good self-care practice (AOR = 3.03, 95% CI 1.10–8.35). Furthermore, the patient from urban areas had higher odds (AOR = 6.47, 95% CI 1.38–30.43) of having good self-care (Table 3).

**Table 3:** Factors affecting the self-care practice among patients with diabetes at Ayder Comprehensive Specialized Hospital, Mekelle, Tigray, Ethiopia, 2017

Variables	Self-care practice			
	Good	Poor	COR, P-value	AOR, P-value
Age in years				
18–35	19 (18.9)	72 (79.1)	1	1
36–50	38 (30.4)	87 (69.6)	1.65 (0.88–3.12), P < 0.12	0.88 (0.31–2.53), P < 0.8
51–65	20 (22.7)	68 (77.3)	1.12 (0.55–2.27), P < 0.77	0.64 (0.20–2.11), P < 0.4
66 and above	10 (29.4)	24 (70.6)	1.58 (0.65–3.86), P < 0.32	0.91 (0.23–3.61), P < 0.9
Sex				
Male	55 (35.9)	129 (74.1)	1	1
Female	32 (20.8)	122 (79.2)	0.62 (0.99–2.68), P < 0.06	1.39 (0.72–2.69), P < 0.33
Level of education				
No formal education	11 (12.1)	80 (87.9)	1	1
Primary school	12 (12.5)	84 (87.5)	1.04 (0.43–2.49), P < 0.93	0.48 (0.17–1.38), P < 0.17
Secondary school	16 (28.6)	40 (71.4)	2.91 (1.24–6.85), P < 0.02	1.25 (0.42–3.74), P < 0.69
Higher school	48 (50.5)	47 (49.5)	7.43 (3.52–15.69), P < 0.00	2.65 (0.95–7.37), p < 0.06**
Type of DM				
Type 1	20 (19.6)	82 (80.4)	1	1
Type 2	67 (28.4)	169 (71.6)	1.63 (0.92–2.86), P < 0.09	1.27 (0.41–3.91), P < 0.68
Religion				
Christian	71 (23.3)	234 (76.7)	1	1
Muslim	16 (48.5)	17 (51.5)	3.10 (1.49–6.45), P < 0.002	3.14 (1.28–7.91), P < 0.02**
Monthly income				
Low	24 (15)	137 (85)	1	1
Medium	39 (37.9)	64 (62.1)	3.48 (1.93–6.27), P < 0.00	1.86 (0.90–3.82), P < 0.09
High	21 (56.8)	16 (43.2)	7.49 (3.43–16.37), P < 0.00	3.03 (1.10–8.35), P < 0.03**
Marital status				
Single	11 (20.4)	43 (79.6)	1	1
Married	66 (29.4)	159 (70.6)	1.62 (0.79–3.34), P < 0.19	1.27 (0.46–3.54), P < 0.65
Divorced	4 (21.1)	15 (78.9)	1.04 (0.29–3.77), P < 0.95	0.87 (0.15–4.92), P < 0.87
Widowed	6 (15)	34 (85)	0.69 (0.23–2.05), P < 0.51	0.86 (0.20–3.73), P < 0.84
Residence				
Urban	84 (31.6)	182 (68.4)	10.61 (3.25–34.70), P < 0.00	6.47 (1.38–30.43), P < 0.02**
Rural	3 (4.2)	69 (95.8)	1	

** Shows statistically significant association

## Discussion

Among the 338 study participants 70.4%, 70.4% and 25.5% of them had a good knowledge, attitude and self-care practices, respectively. Being male, widowed, living in urban, and earning medium income was significantly associated with better knowledge of self-care. Having a higher income was significantly associated with a good attitude towards diabetic self-care. On the contrary, taking both insulin and oral hypoglycemic together was associated with a poor attitude. Being Muslim, living in urban areas and earning a high income were determinants of good self-care practice.

The majority of (70.4%) of the study participants were found to have good knowledge which is comparable to the result of a study done in Adama, Ethiopia (77.6%) [15]. But it is higher than a study conducted in Egypt (52.3%) [16]. The relatively high number of knowledgeable patients in this study could be explained by the higher percentage of educated participants and the difference in training received at the diabetic clinic. One study done in Addis Ababa, Ethiopia indicated that high school and diabetes education attendance had a significant influence on the knowledge of diabetes [17].

Male patients were more likely to have a good knowledge of diabetes self-care. The finding was in accordance with studies done in Egypt, Bangladesh and UAE [8, 16, 18]. This higher knowledge level of male patients could be due to the fact that they are more likely to be educated and likely to go to diabetic clinics. Therefore, they will have fewer barriers in communicating with the health care teams.

Patients who live in urban were more likely to have a good knowledge of diabetes self-care. Similar findings reported in Egypt [16]. This association could be due to more opportunities for exposure to information about diabetes self-care through the mass media, books and internet in urban areas.

Among the respondents, 70.4% had a good attitude towards performing self-care practices. This is slightly lower than studies done in South Africa and Ethiopia, where 84.3% and 81.9% of the participants having a positive attitude towards life style modifications, respectively [7, 15]. On the contrary, a study done in Pakistan revealed that most participants had a negative attitude regarding diabetes [19] this variation could be due to utilization of different tools for data collection. Patients who were taking both insulin and oral hypoglycemic agents had a poor attitude towards self-care. It is likely that this poor attitude leads them to poor glycemic control and that’s why they are taking both insulin and oral hypoglycemic agents.

About three quarter (74.5%) of the study participants had poor self-care practice. This is higher than the study done in other parts of the country with 45%, 55% and 60.7% of the participants had poor self-care practice in Nekemt, Jimma and Harari, respectively [9, 13, 14]. In Adama 33.6% of the respondents were with low life style modification practice [15]. Another study conducted in Kenya showed that, 59% of the participants had poor self-care practices [20]. About half (47.6%) of the patients had low monthly income below 1000 ETB this could limit their accessibility and affordability of a well-balanced diet. the difference in techniques used, differences in educational background and strength of diabetic associations and implementation of its principles in the study area may contribute to the variation.

The findings of multivariate regression analysis showed that being Muslim, living in urban areas, having a high income and higher educational status were significantly associated with good self-care practice. The reason behind may be because of fewer barriers in communicating with the health care teams and more opportunities for exposure to information about diabetes self-care through the mass media, books and internet respectively. Living in rural areas may hinder practicing the recommended activities by limiting access to and affordability to a well-balanced diet and healthy food [17].

## Conclusion

The level of self-care practice in patients with diabetes was found to be sub-optimal even though the majority of them had a good knowledge and attitude. An effort from all concerned bodies should increase in closing the gap between knowledge and practice. The study findings would draw the attention of practitioners in closing the gap between knowledge and practice of self-care among patients with diabetes. Health care providers should be empowered for delivering adequate health message regarding diabetic self-care practices.

## Limitations

The results may not reflect the actual knowledge, attitude and practice of patients with diabetes because of two reasons. One, there may be recall bias by the patients during data collection. In addition, the patients may have provided socially acceptable responses.

## Additional files

**Additional file 1.** English version Questionnaires: sociodemographic, clinical, knowledge, attitude and practice questions.

**Additional file 2.** SPSS data set.

**Additional file 3: Table S1.** Socio demographic and clinical characteristics of patients with diabetes at Ayder Comprehensive Specialized Hospital, Mekelle, Tigray, Ethiopia, 2017.

**Additional file 4: Table S2.** Status of knowledge among patients with diabetes at Ayder Comprehensive Specialized Hospital, Mekelle, Tigray, Ethiopia, 2017.

**Additional file 5: Figure S1.** Attitude status towards diabetic self-care among patients with diabetes at Ayder Comprehensive Specialized Hospital, Mekelle, Tigray, Ethiopia 2017.

**Additional file 6: Table S3.** Status of self-care practice among patients with diabetes at Ayder Comprehensive Specialized Hospital, Mekelle, Tigray, Ethiopia, 2017.

## Abbreviations

AOR: adjusted odds ratio
CI: confidence interval
DAS3: Diabetes Attitude Survey 3
DM: diabetes mellitus
IDF: International Diabetes Federation
SDSCA: summary of diabetes self-care activities
SKILLDS: Spoken Knowledge in Low Literacy in Diabetes Scale
