# Supplementary material for: Self-care related knowledge, attitude, practice and associated factors among patients with diabetes in Ayder Comprehensive Specialized Hospital, North Ethiopia
Source: BMC Res Notes. 2019 Jan 18;12:34. doi: 10.1186/s13104-019-4072-z (PMC6339268; doi:10.1186/s13104-019-4072-z)
Supplement: Supplementary file 1 — Additional file 1. English version Questionnaires: sociodemographic, clinical, knowledge, attitude and practice questions. [file 13104_2019_4072_MOESM1_ESM.docx]

## English version Questionnaires

Part one: Socio demographic characteristics of patients

Age (years)

Sex Male Female

Marital status Single Married Divorced Widowed

Family size ___________

Religion Orthodox Muslim Catholic

Protestant other specify_____

Occupation

Business man Governmental organization worker farmer

Private organization worker unemployed
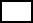
 retried Student

others specify__________________

Residence Urban Rural

Educational status

No formal education Primary school Secondary school Higher education

Monthly income (ETB) _________________________

Part two: Disease and treatment related questions

How long have you been since you diagnosed as DM? (in years) ______

What type of diabetes do you have? Type 1
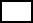
 Type 2
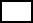
 others:_____

How long have you been on anti-diabetic medications? (in years) ________

Currently what treatments are you receiving for diabetes prescribed by a health care worker?

Insulin injection Oral anti-diabetic drugs TLC

What other comorbidities do you have_?_

Hypertension dyslipidemia cardiovascular disease if other specify: _________

Part Three: Questions on knowledge of self-care practices

1. What are the signs and symptoms of High Sugar? If no answer: How do you feel when your blood sugar is high?

At least 2 correct answer.

Frequent urination, Extreme thirst or eating a lot, blurred vision, drowsiness, fatigue.

2. What are the signs and symptoms of low sugar? How do you feel when your blood sugar is too low?

At least 2 correct answer.

Hunger, nervousness, restlessness, confusion, sweaty, fast heart rate.

3. How do you treat low blood sugar? If no Answer: What should you do if your sugar is too low?

At least one correct answer. Juice, milk, hard candy, sugar

4. How often should a person with diabetes check his or her feet? If no answer: Once a day, once a week or once a month?

Correct answer. Daily

5. Why are feet exams important in someone with diabetes? If no answer: Why is it important to look at your feet?

Accept very general answer. Prevention of morbidity due to neuropathic/immunological

Consequences of diabetes.

6. How often should you see your eye doctor and why is this important?

Correct answer. Seen at least yearly AND prevent retinopathy (blindness).

7. How many times per week should someone with diabetes exercise and for how long?

Correct answer. 3 -5 times a week for a total of 30minute and above.

8. What are some of the long term complications of uncontrolled diabetes?

Needs at least 2.

Blindness, kidney disease, amputation, neuropathy, cardiovascular disease.

Part four: Diabetic attitude questioner

1. How often should self-care management practiced.

1. Often 2. Sometimes 3. Rarely 4. After a damage occurred 5. No need to practice

2. I can prevent the complications of diabetes mellitus by using self-care practices appropriately.

1. Strongly disagree 2. Disagree 3. Neutral 4. Agree 5. Strongly agree

3. The support from family and friends is important in self-care practice.

1. Strongly disagree 2. Disagree 3. Neutral 4. Agree 5. Strongly agree

4. Diabetic patients are responsible in addition to their doctor and family in the care of diabetes.

1. Strongly disagree 2. Disagree 3. Neutral 4. Agree 5. Strongly agree

5. It’s my responsibility to do regular checkups to ophthalmologist.

1. Strongly disagree 2. Disagree 3. Neutral 4. Agree 5. Strongly agree

6. If I see a problem in my feet I report to my health provider immediately.

1. Strongly disagree 2. Disagree 3. Neutral 4. Agree 5. Strongly agree

7. Self-care practices are as effective as medication for my condition if followed properly.

1. Strongly disagree 2. Disagree 3. Neutral 4. Agree 5. Strongly agree

Part five: Diabetes self-care practice

The questions below ask you about your diabetes self-care activities during the past 7 days. If you were sick during the past 7 days, please think back to the 7 days that you were not sick.

|  | Diet |  | Number of days | | | | | |  | remark |
| --- | --- | --- | --- | --- | --- | --- | --- | --- | --- | --- |
|  |  | 0 | 1 | 2 | 3 | 4 | 5 | 6 | 7 |  |
| 1 | How many of the last SEVEN DAYS have you followed a healthful eating plan? |  |  |  |  |  |  |  |  |  |
| 2 | On average over the past month, how many DAYS PER WEEK have you followed your eating plan? |  |  |  |  |  |  |  |  |  |
| 3 | On how many of the last SEVEN DAYS did you eat five or more servings of fruits and vegetables? |  |  |  |  |  |  |  |  |  |
| 4 | On how many of the last SEVEN DAYS did you eat high fat foods Such as red meat or full fat dairy products? |  |  |  |  |  |  |  |  |  |
| 5 | On how many of the last SEVEN DAYS did you space carbohydrates evenly through the day? |  |  |  |  |  |  |  |  |  |
|  | Physical Activity |  |  |  |  |  |  |  |  |  |
| 6 | On how many of the last SEVEN DAYS did you participate in a specific exercise session other than what you do around the house or as part of your work? |  |  |  |  |  |  |  |  |  |
|  | Blood sugar testing |  |  |  |  |  |  |  |  |  |
| 7 | On how many of the last SEVEN DAYS did you test your blood sugar?  By Self 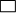 Healthcare provider recommendation 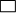 |  |  |  |  |  |  |  |  |  |
|  | Foot care |  |  |  |  |  |  |  |  |  |
| 8 | On how many of the last SEVEN DAYS did you check your feet? |  |  |  |  |  |  |  |  |  |
| 9 | On how many of the last SEVEN DAYS did you inspect the inside of your shoes? |  |  |  |  |  |  |  |  |  |
|  | Medication |  |  |  |  |  |  |  |  |  |
| 10 | On how many of the last SEVEN DAYS did you take your recommended insulin injections? |  |  |  |  |  |  |  |  |  |
| 11 | On how many of the last SEVEN DAYS did you take your recommended number of diabetes pills? |  |  |  |  |  |  |  |  |  |
|  | Smoking |  |  |  |  |  |  |  |  |  |
| 12 | Have you smoked a cigarette, even a puff in the past SEVEN DAYS? |  |  |  |  |  |  |  |  |  |
| 13 | Have you drank Alcohol in the past SEVEN DAYS? |  |  |  |  |  |  |  |  |  |
