# Supplementary material for: Self-care related knowledge, attitude, practice and associated factors among patients with diabetes in Ayder Comprehensive Specialized Hospital, North Ethiopia
Source: BMC Res Notes. 2019 Jan 18;12:34. doi: 10.1186/s13104-019-4072-z (PMC6339268; doi:10.1186/s13104-019-4072-z)
Supplement: Supplementary file 3 — Additional file 3: Table S1. Socio demographic and clinical characteristics of patients with diabetes at Ayder Comprehensive Specialized Hospital, Mekelle, Tigray, Ethiopia, 2017. [file 13104_2019_4072_MOESM3_ESM.docx]

**Table S1: Socio demographic and clinical characteristics of patients with diabetes at Ayder Comprehensive Specialized Hospital, Mekelle, Tigray, Ethiopia, 2017 (N=338).**

| **Variables** | **Number (%)** |
| --- | --- |
| Gender |  |
| Male  Female | 184(54.4)  154(45.6) |
| Age (years) (Mean ± SD) (45.78± 14.72)  18-35  36-50  51-65  66 and above | 91(26.9)  125 (37.0m)  88(26.0)  34(10.1) |
| Level of Formal Education |  |
| No formal education  Primary school education  Secondary school education  Higher education | 91(26.9)  96 (28.4)  56(16.6)  95(28.1) |
| Religion  Orthodox  Muslim  Protestant  others | 302(89.3)  33(9.8)  2(0.6)  1(0.3) |
| Residence  Urban  Rural | 266(78.7)  72(21.3) |
| Level of monthly income(ETB) |  |
| Low (<= 1000)  Medium (1001-4000)  High (>4000)  Unknown | 161(47.6)  103(30.5)  37(10.9)  37(10.9) |
| Duration of illness(years) |  |
| <5 years  6 - 10 years  11-15 years  16 years and above | 166(49.1)  91(26.9)  54(16.0)  27(8.0) |
| Duration of anti-diabetic use  (Mean ± SD) (6.92±5.48)  <5 years  6 - 10 years  11-15 years  16 and above | 171(50.6)  90(26.6)  51(15.1)  26(7.7) |
| Type of diabetes  Type 1  Type 2 | 102(30.2)  236(69.8) |
| Treatment modality  Insulin only  Oral anti diabetic agents only  Oral anti-diabetic agents and insulin | 181(53.6)  148(43.8)  9(2.7) |
