# Supplementary material for: Self-care related knowledge, attitude, practice and associated factors among patients with diabetes in Ayder Comprehensive Specialized Hospital, North Ethiopia
Source: BMC Res Notes. 2019 Jan 18;12:34. doi: 10.1186/s13104-019-4072-z (PMC6339268; doi:10.1186/s13104-019-4072-z)
Supplement: Supplementary file 4 — Additional file 4: Table S2. Status of knowledge among patients with diabetes at Ayder Comprehensive Specialized Hospital, Mekelle, Tigray, Ethiopia, 2017. [file 13104_2019_4072_MOESM4_ESM.docx]

**Table S2: Status of knowledge among patients with diabetes at Ayder Comprehensive Specialized Hospital, Mekelle, Tigray, Ethiopia, 2017.**

| Knowledge of diabetic patients on self-care | Correct response  Frequency (%) |
| --- | --- |
| What are the signs and symptoms of High Sugar? | 202 (59.8) |
| What are the signs and symptoms of low sugar? | 109 (32.2) |
| How do you treat low blood sugar? | 308 (91.2) |
| How often should a person with diabetes check his or her feet? | 177 (52.4) |
| Why are feet exams important in someone with diabetes? | 214 (63.3) |
| How often should you see your eye doctor? | 80 (23.7) |
| Why it is important to have eye exam? | 202 (59.8) |
| How many times per week should someone with diabetes exercise | 271 (80.2 ) |
| For how long should someone with diabetes exercise? | 260 (76.9) |
| What are some of the long term complications of uncontrolled diabetes? | 134 (39.6 |
| Total | 238 (70.4) |
