# Supplementary material for: Self-care related knowledge, attitude, practice and associated factors among patients with diabetes in Ayder Comprehensive Specialized Hospital, North Ethiopia
Source: BMC Res Notes. 2019 Jan 18;12:34. doi: 10.1186/s13104-019-4072-z (PMC6339268; doi:10.1186/s13104-019-4072-z)
Supplement: Supplementary file 5 — Additional file 5: Figure S1. Attitude status towards diabetic self-care among patients with diabetes at Ayder Comprehensive Specialized Hospital, Mekelle, Tigray, Ethiopia 2017. [file 13104_2019_4072_MOESM5_ESM.docx]

**Figure S1: Attitude status towards diabetic self-care among patients with diabetes at Ayder Comprehensive Specialized Hospital, Mekelle, Tigray, Ethiopia 2017.**
