# Supplementary material for: Self-care related knowledge, attitude, practice and associated factors among patients with diabetes in Ayder Comprehensive Specialized Hospital, North Ethiopia
Source: BMC Res Notes. 2019 Jan 18;12:34. doi: 10.1186/s13104-019-4072-z (PMC6339268; doi:10.1186/s13104-019-4072-z)
Supplement: Supplementary file 6 — Additional file 6: Table S3. Status of self-care practice among patients with diabetes at Ayder Comprehensive Specialized Hospital, Mekelle, Tigray, Ethiopia, 2017. [file 13104_2019_4072_MOESM6_ESM.docx]

| Variable | Good  N (%) | Poor  N (%) |
| --- | --- | --- |
| Adherence to a healthful eating plan | 140(41.4) | 198(58.6) |
| Adherence to physical activity | 159(47) | 179(53) |
| Adherence for testing of blood glucose | 18(5.3) | 320(94.7) |
| Adherence for foot check | 160(47.3) | 178(52.7) |
| Adherence for taking recommended insulin injections | 305(90.2) | 33(9.8) |
| Adherence for taking recommended number of diabetes pills | 321(95.0) | 17(5.0) |
| Total practice score | 81(25.5) | 257(74.5) |

**Table S3: status of self-care practice among patients with diabetes at Ayder Comprehensive Specialized Hospital, 2017.**
